# *In vitro* and *in vivo* growth inhibitory activities of cryptolepine hydrate against several *Babesia* species and *Theileria equi*

**DOI:** 10.1371/journal.pntd.0008489

**Published:** 2020-08-27

**Authors:** Gaber El-Saber Batiha, Amany Magdy Beshbishy, Luay M. Alkazmi, Eman H. Nadwa, Eman K. Rashwan, Naoaki Yokoyama, Ikuo Igarashi

**Affiliations:** 1 National Research Center for Protozoan Diseases, Obihiro University of Agriculture and Veterinary Medicine, Obihiro, Hokkaido, Japan; 2 Department of Pharmacology and Therapeutics, Faculty of Veterinary Medicine, Damanhour University, Damanhour, Albeheira, Egypt; 3 Biology department, Faculty of Applied Sciences, Umm Al-Qura University, Makkah, Saudi Arabia; 4 Department of Pharmacology and Therapeutics, College of Medicine, Jouf University, Sakaka, Saudi Arabia; 5 Department of Medical Pharmacology, Faculty of Medicine, Cairo University, Giza, Egypt; 6 Department of Physiology, College of Medicine, Al-Azhar University, Assuit, Egypt; 7 Department of Physiology, College of Medicine, Jouf University, Sakaka, Saudi Arabia; Johns Hopkins Bloomberg School of Public Health, UNITED STATES

## Abstract

Piroplasmosis treatment has been based on the use of imidocarb dipropionate or diminazene aceturate (DA), however, their toxic effects. Therefore, the discovery of new drug molecules and targets is urgently needed. Cryptolepine (CRY) is a pharmacologically active plant alkaloid; it has significant potential as an antiprotozoal and antibacterial under different *in vitro* and *in vivo* conditions. The fluorescence assay was used for evaluating the inhibitory effect of CRY on four *Babesia* species and *Theileria equi in vitro*, and on the multiplication of *B*. *microti* in mice. The toxicity assay was evaluated on Madin–Darby bovine kidney (MDBK), mouse embryonic fibroblast (NIH/3T3), and human foreskin fibroblast (HFF) cell lines. The half-maximal inhibitory concentration (IC_50_) values of CRY on *Babesia bovis*, *B*. *bigemina*, *B*. *divergens*, *B*. *caballi*, and *T*. *equi* were 1740 ± 0.377, 1400 ± 0.6, 790 ± 0.32, 600 ± 0.53, and 730 ± 0.025 nM, respectively. The toxicity assay on MDBK, NIH/3T3, and HFF cell lines showed that CRY affected the viability of cells with a half-maximum effective concentration (EC_50_) of 86.67 ± 4.43, 95.29 ± 2.7, and higher than 100 μM, respectively. In mice experiments, CRY at a concentration of 5 mg/kg effectively inhibited the growth of *B*. *microti*, while CRY–atovaquone (AQ) and CRY–DA combinations showed higher chemotherapeutic effects than CRY alone. Our results showed that CRY has the potential to be an alternative remedy for treating piroplasmosis.

## Author summary

The development and evaluation of new treatment strategies against *Babesia* and *Theileria* parasites have become increasingly urgent due to the emergence of parasite resistance and unwanted toxicity side effects by current chemotherapies. On the other hand, vaccination is a cheaper, reliable and sustainable option. Unfortunately, it has not worked well for the protozoan diseases because they possess ingenious mechanisms to evade the host immune system, rendering treatment the most suitable approach for their control. Sadly, only diminazene aceturate (DA) and imidocarb dipropionate have passed clinical trials for the treatment of piroplasmosis. However, these drugs cause many adverse effects and not approved yet for human medicine. Cryptolepine (CRY) is a pharmacologically active indoloquinoline alkaloid isolated from the roots of the shrub *Cryptolepis sanguinolenta*. CRY is reported to possess various pharmacological activities, including antifungal, anti-mycobacterial, and potent antiplasmodial activities. In the present study we evaluated the effects of CRY against the growth of *Babesia bovis*, *B*. *bigemina*, *B*. *divergens*, *B*. *caballi* and *Theileria equi in vitro* and its chemotherapeutic potential on *Babesia microti* in mice. Furthermore, we investigated the effect of combination between CRY with the current babesiocidal drugs such as DA, atovaquone (AQ) and clofazimine (CF) *in vitro* and *in vivo*.

## Introduction

Despite huge efforts and resources to eradicate piroplasmosis, the disease remains a serious productivity problem, costing hundreds of thousands of dollars annually. Attempts are being made to develop vaccines against bovine and equine piroplasmosis, but progress has been slow [1], therefore, drug therapy is considered the main tool for controlling piroplasmosis. Diminazene aceturate (DA) and imidocarb dipropionate are still the main choices for treating bovine and equine piroplasmosis in spite of their toxic side effects [2] and the development of DA-resistant *Babesia gibsoni* and imidocarb dipropionate–resistant *Theileria equi* [3, 4]. Moreover, Zintl et al. documented therapeutic failures in some severe cases infected with human babesiosis after the administration of clindamycin, azithromycin, quinine, and tetracycline [5]. Therefore, the development of the above-mentioned problems encouraged us to search for chemical compounds with novel structures and natural origins that represent new and distinct chemical classes and mechanisms of actions from those of currently available antipiroplasmic drugs [2, 6].

Cryptolepine (CRY) is a pharmacologically active indoloquinoline alkaloid isolated from the roots of the shrub *Cryptolepis sanguinolenta* [7]. CRY is reported to possess various pharmacological activities, including antihyperglycemic [8], antifungal [9], antihypertensive [10], anti-mycobacterial [11], anticancer [7], and potent antiplasmodial activities against both chloroquine-resistant and -sensitive strains [12, 13]. Recently, CRY has been considered a remarkable leading compound in the search for effective and new antimalarial drugs [13]. Although the molecular basis for its diverse biological effects remains largely ambiguous, mechanistic studies have shown that CRY acts through several mechanisms. For instance, Chong and Sullivan. and Lavrado et al. documented that the antiplasmodial effect of CRY is associated with the inhibition of hemozoin formation, which eventually leads to parasite death [14, 15]. Additionally, Olajide et al. reported that the anti-inflammatory action of CRY is mediated through both inflammatory mediator signaling (COX-2/PGE2) and inflammatory promoters (TNFα and iNOS) [16, 17]. Pal and Katiyar. revealed that CRY’s anticancer effects were due to its direct interaction with DNA [18]. Sawer et al. reported that the antibacterial as well as the antifungal activity of CRY was attributed to disturbance of surface structure that lead to cellular breakdown and morphological changes in *Staphylococcus aureus*, *Saccharomyces cerevisiae* and *Candida albicans* [5]. Cryptolepine is also known to be a DNA intercalator and an inhibitor of DNA synthesis through topoisomerase inhibition. Competition dialysis assays demonstrated that CRY is able to bind CG-rich sequences containing non-alternating cytosine-cytosine sites [19].

Our study’s rationale was aimed at looking for new antipiroplasmic compounds to address the challenges presented by currently available drugs (diminazine aceturate and imidocarb dipropionate), such as the risk of toxicity, drug-resistant parasites, drug residues and withdrawal problems, which impede the use of these drugs for the treatment of equine and bovine piroplasmosis in many countries. As well as looking for lead compounds that could pave the way for discovering and recognizing some analogues with high efficacy and low toxic effects. Therefore, the current study aimed to assess the effectiveness of CRY, the well-known antiplasmodial drug, against the growth of bovine *Babesia* (*B*. *bovis*, *B*. *bigemina*, and *B*. *divergens*) and equine piroplasm parasites (*B*. *caballi* and *T*. *equi*) using an *in vitro* culture. It also evaluated the chemotherapeutic effect of CRY on rodent *Babesia* that also infects humans (*B*. *microti*) using a mouse model. Furthermore, we investigated the effect of combination treatment of CRY with the current babesiocidal drugs such as DA, atovaquone (AQ), and clofazimine (CF) on the *in vitro* growth of *B*. *bovis*, *B*. *bigemina*, *B*. *divergens*, *B*. *caballi*, and *T*. *equi*, as well as their chemotherapeutic activities against *B*. *microti* in mice.

## Results

### The growth inhibitory effect of CRY against *Babesia* and *Theileria in vitro*

**Fig 1:**
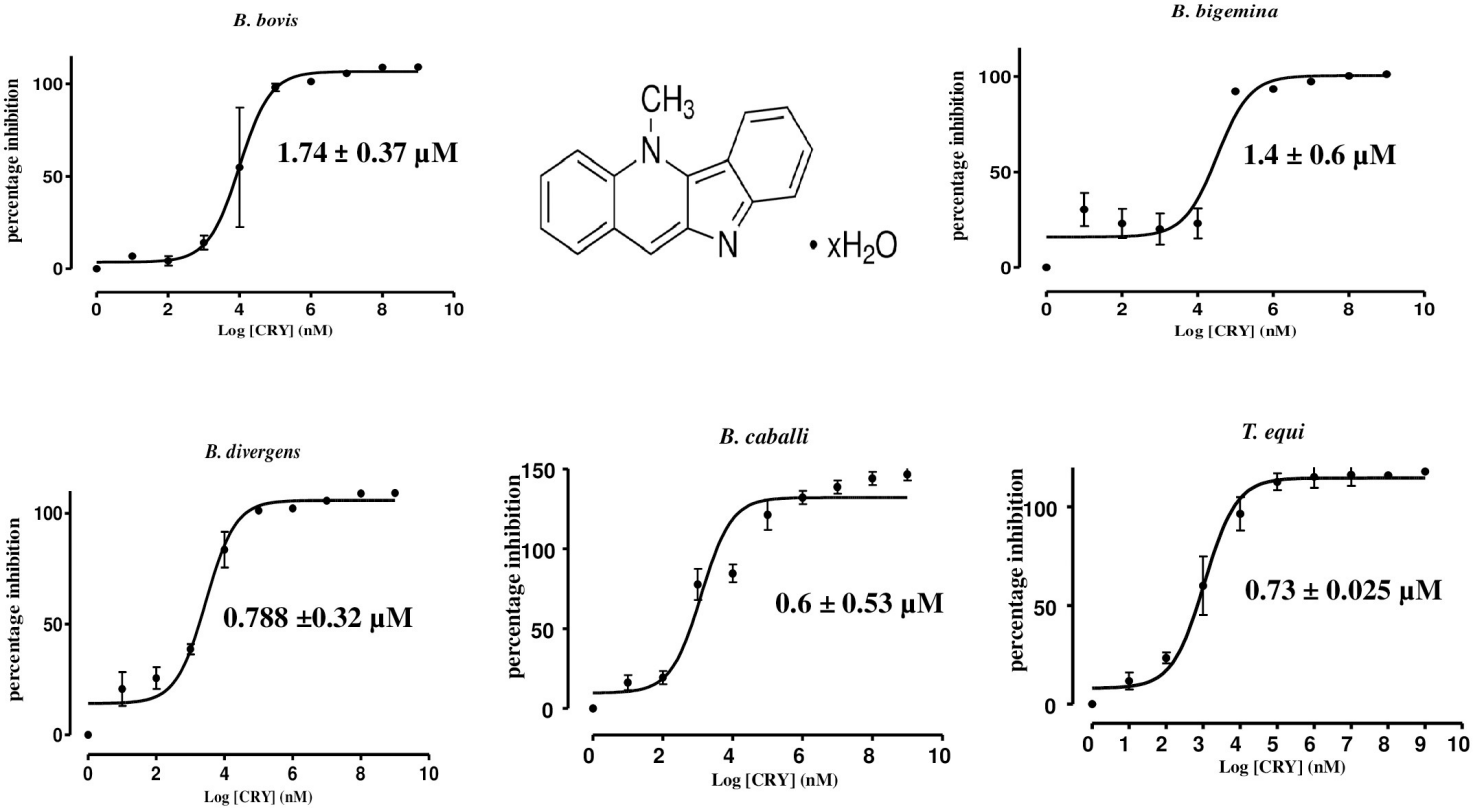
The dose-response curves of CRY against *Babesia* and *Theileria* parasites *in vitro*. The curves show the growth inhibition of *B*. *bovis*, *B*. *bigemina*, *B*. *divergens*, *B*. *caballi*, and *T*. *equi* treated with various concentrations of CRY. The result was determined by fluorescence assay after 96 h of incubation. The values obtained from three separate trials were used to determine the IC_50_s using nonlinear regression (curve fitting analysis) in GraphPad Prism software (GraphPad Software Inc., USA).

The growth inhibitory assay was carried out on four *Babesia* species, namely, *B*. *bovis*, *B*. *bigemina*, *B*. *divergens* and *B*. *caballi*, and *T*. *equi*. CRY inhibited the multiplication of all species in a dose-dependent manner. CRY significantly inhibited the *in vitro* growth of *B*. *bovis* and *B*. *bigemina* (t-test: *t*_(9)_ = 2.782, *P* = 0.005) at 0.15 μM and significantly inhibited the growth of *B*. *divergens* (t-test: *t*_(9)_ = 3.954, *P* = 0.003) and *T*. *equi* (t-test: *t*_(9)_ = 2.549, *P* = 0.02) at 0.078 μM. The *in vitro* growth of *B*. *caballi* was significantly inhibited (t-test: *t*_(9)_ = 4.464, *P* = 0.001) with 0.03 μM treatment of CRY (Fig 1).

The IC_50_ values of CRY on *B*. *bovis*, *B*. *bigemina*, *B*. *divergens*, *B*. *caballi*, and *T*. *equi* were 1740 ± 0.377, 1400 ± 0.6, 790 ± 0.32, 600 ± 0.53, and 730 ± 0.025 nM, respectively (Table 1). Meanwhile, DA showed IC_50_ values at 350 ± 0.06, 680 ± 0.09, 430 ± 0.05, 22 ± 0.0002, and 710 ± 0.05 nM against *B*. *bovis*, *B*. *bigemina*, *B*. *divergens*, *B*. *caballi*, and *T*. *equi*, respectively (Table 1).

**Table 1:** The IC_50_ and selectivity index of CRY and DA.

Compound	Parasite	IC_50_ (nM)^a^	EC_50_ (μM)^b^			Selective index^c^		
			MDBK	NIH/3T3	HFF	MDBK	NIH/3T3	HFF
**CRY**	*B*. *bovis*	**1740 ± 0.37**	**86.67±4.43**	**95.29±2.7**	**˃100**	**49.81**	**57.77**	**˃ 57.5**
	*B*. *bigemina*	**1400 ± 0.6**	**86.67±4.43**	**95.29±2.7**	**˃100**	**61.91**	**68.07**	**˃ 71.4**
	*B*. *divergens*	**790 ± 0.32**	**86.67±4.43**	**95.29±2.7**	**˃100**	**109.71**	**120.62**	**˃ 126.9**
	*B*. *caballi*	**600 ± 0.53**	**86.67±4.43**	**95.29±2.7**	**˃100**	**144.45**	**158.82**	**˃ 166.7**
	*T*. *equi*	**730 ± 0.025**	**86.67±4.43**	**95.29±2.7**	**˃100**	**118.73**	**130.54**	**˃ 136.9**
**DA**	*B*. *bovis*	**350 ± 0.06**	**˃100**	**˃100**	**˃100**	**˃ 285.7**	**˃ 285.7**	**˃ 285.7**
	*B*. *bigemina*	**680 ± 0.09**	**˃100**	**˃100**	**˃100**	**˃ 147.1**	**˃ 147.1**	**˃ 147.1**
	*B*. *divergens*	**430 ± 0.05**	**˃100**	**˃100**	**˃100**	**˃ 232.5**	**˃ 232.5**	**˃ 232.5**
	*B*. *caballi*	**20 ± 0.0002**	**˃100**	**˃100**	**˃100**	**˃ 5000**	**˃ 5000**	**˃ 5000**
	*T*. *equi*	**710 ± 0.05**	**˃100**	**˃100**	**˃100**	**˃ 140.8**	**˃ 140.8**	**˃ 140.8**

^a^Half-maximal inhibition concentration of cryptolepine (CRY) and diminazene aceturate (DA) on the *in vitro* culture of parasites. The value was determined from the dose-response curve using nonlinear regression (curve fit analysis). The values are the means of triplicate experiments.

^b^Half-maximal effective concentration of CRY and DA on cell lines. The values were determined from the dose-response curve using nonlinear regression (curve fit analysis). The values are the means of triplicate experiments.

^c^Ratio of the EC_50_ of cell lines to the IC_50_ of each species. High numbers are favorable.

**Fig 2:**
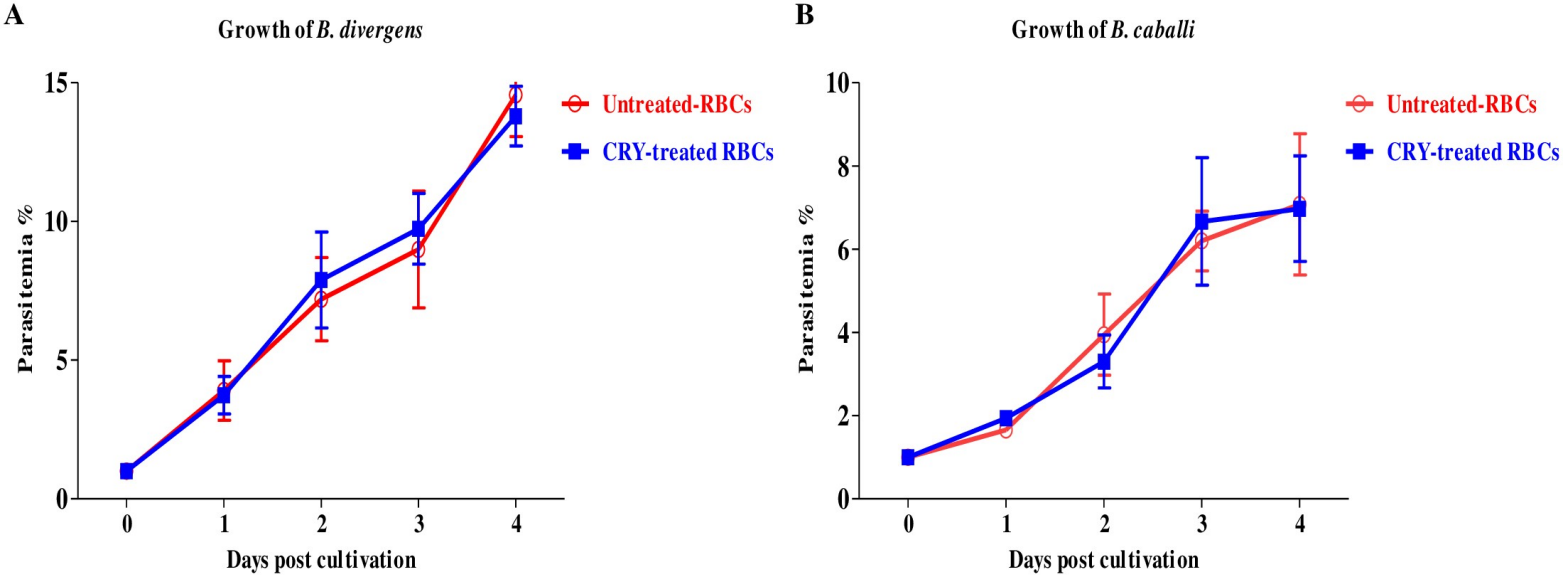
Growth of *B*. *divergens* with CRY-treated bovine RBCs (A) and growth of *B*. *caballi* with CRY-treated horse RBCs (B).

AQ and CF inhibited the growth of all tested parasites with IC_50_ values shown in S1 Table. Compared with other drugs tested on the present study, CRY showed a similar inhibitory effect of DA against *B*. *divergens* and *T*. *equi*, while it was more effective than CF against all tested parasites. Since there was no significant difference in the inhibition between wells containing DMSO and untreated wells, the effectiveness of CRY was not influenced by the diluent. The pre cultivation of cattle and horse RBCs with CRY was carried out to determine its direct effect on the host RBCs. Microscopy observation and parasitemia counting showed that the multiplication of *B*. *divergens* (Fig 2A) and *B*. *caballi* (Fig 2B) did not significantly differ between CRY-treated RBCs and normal RBCs for either species.

### Toxicity of CRY, DA, CF, and AQ on NIH/3T3, HFF, and MDBK cell lines

CRY showed effective inhibition on the *in vitro* culture of *Babesia* and *Theileria* parasites higher than that of currently available drug (CF). Thus, the cytotoxicity of CRY was evaluated using NIH/3T3, HFF, and MDBK cell lines to see its effect on the viability of host cells (Table 1). At a concentration of 100 μM, CRY did not show any inhibition on HFF cell line, while it showed inhibition only on MDBK and NIH/3T3 cell lines with EC_50_ values at 86.67 ± 4.43 and 95.29 ± 2.7 μM, respectively (Table 1). In a separate assay, DA (Table 1) as well as AQ did not show any inhibition on NIH/3T3, HFF, or MDBK cell line at a concentration of 100 μM, while CF showed inhibition only on MDBK cell line with EC_50_ value at 34 ± 3.4 μM (S1 Table), whereas the selectivity indexes are defined as the ratio of EC_50_ of the tested compounds on the cell line to IC_50_ of these compounds on the parasite. For CRY, in the cases of HFF, NIH/3T3, and MDBK cell lines, the highest selectivity index was found to be >166.7, 158.82, and 144.45 times higher than the IC_50_, respectively on *B*. *caballi* (Table 1).

### The effects of the combination of CRY with DA, CF, and AQ *in vitro*

CompuSyn software was used to analyze the percentage of inhibition of the single drug and each combination to generate the combination index (CI) values (Table 2). Combination treatments of CRY with DA showed an additive effect against *B*. *bovis*, *B*. *caballi*, and *T*. *equi*, while showed a moderate antagonistic effect against *B*. *bigemina* and *B*. *divergens*. Combination treatments of CRY with CF showed an additive effect against *B*. *bovis*, *B*. *bigemina* and *B*. *divergens*, while showed a moderate antagonistic effect against *B*. *caballi* and *T*. *equi*. Combination treatments of CRY with AQ showed a synergetic effect against *B*. *bovis*, *B*. *divergens*, and *B*. *caballi*, while showed an additive effect against *B*. *bigemina* and *T*. *equi* (Table 2).

**Table 2:** The effect of CRY with DA, AQ, and CF against *Babesia* and *Theileria* parasites *in vitro*.

Parasite	Drug combination^a^	CI values				Weighted average CI values^b^	Degree of association^c^
		IC_50_	IC_75_	IC_90_	IC_95_		
***B*. *bovis***	**CRY + DA**	0.918	1.071	1.007	1.116	1.0540	**Additive**
	**CRY + AQ**	0.320	0.611	0.222	0.368	0.3680	**Synergistic**
	**CRY + CF**	0.599	1.398	1.123	1.117	1.1230	**Additive**
***B*. *bigemina***	**CRY + DA**	1.897	0.914	1.733	0.914	1.2580	**Moderate antagonism**
	**CRY + AQ**	1.652	1.000	1.000	1.091	1.1016	**Additive**
	**CRY + CF**	1.219	1.099	0.771	0.873	0.9222	**Additive**
***B*. *divergens***	**CRY + DA**	1.798	1.192	1.535	1.512	1.4535	**Moderate antagonism**
	**CRY + AQ**	0.721	0.773	0.731	0.752	0.7468	**Synergistic**
	**CRY + CF**	1.121	0.994	1.479	0.867	1.1014	**Additive**
***B*. *caballi***	**CRY + DA**	1.002	1.010	1.090	1.068	1.0564	**Additive**
	**CRY + AQ**	0.981	0.955	0.771	0.978	0.8098	**Synergistic**
	**CRY + CF**	1.152	1.373	1.777	1.434	1.3813	**Moderate antagonism**
***T*. *equi***	**CRY + DA**	0.983	1.169	0.793	0.932	0.9428	**Additive**
	**CRY + AQ**	1.100	1.020	1.009	1.005	1.0087	**Additive**
	**CRY + CF**	1.932	1.999	0.901	1.087	1.2981	**Moderate antagonism**

CI value, combination index value; IC_50_, 50% inhibition concentration; DA, diminazene aceturate; CF, clofazimine; AQ, atovaquone.

^a^Two-drug combination between CRY with diminazene aceturate, atovaquone, and clofazimine at concentrations of approximately 0.25 x IC_50_, 0.5 x IC_50_, IC_50_, 2 x IC_50_, and 4 x IC_50_ (constant ratio).

^b^The higher inhibition is preferable, thus the weighted average CI value was calculated with the formula [(1 x IC_50_) + (2 x IC_75_) + (3 x IC_90_) + (4 x IC_95_)]/10.

^c^The degree of synergism was determined based on the following CI value: < 0.90 (synergistic), 0.90–1.10 (additive), 1.20–1.45 (moderate antagonism), and > 1.50 (antagonistic).

### The chemotherapeutic effect of CRY against *B*. *microti* in mice

**Fig 3:**
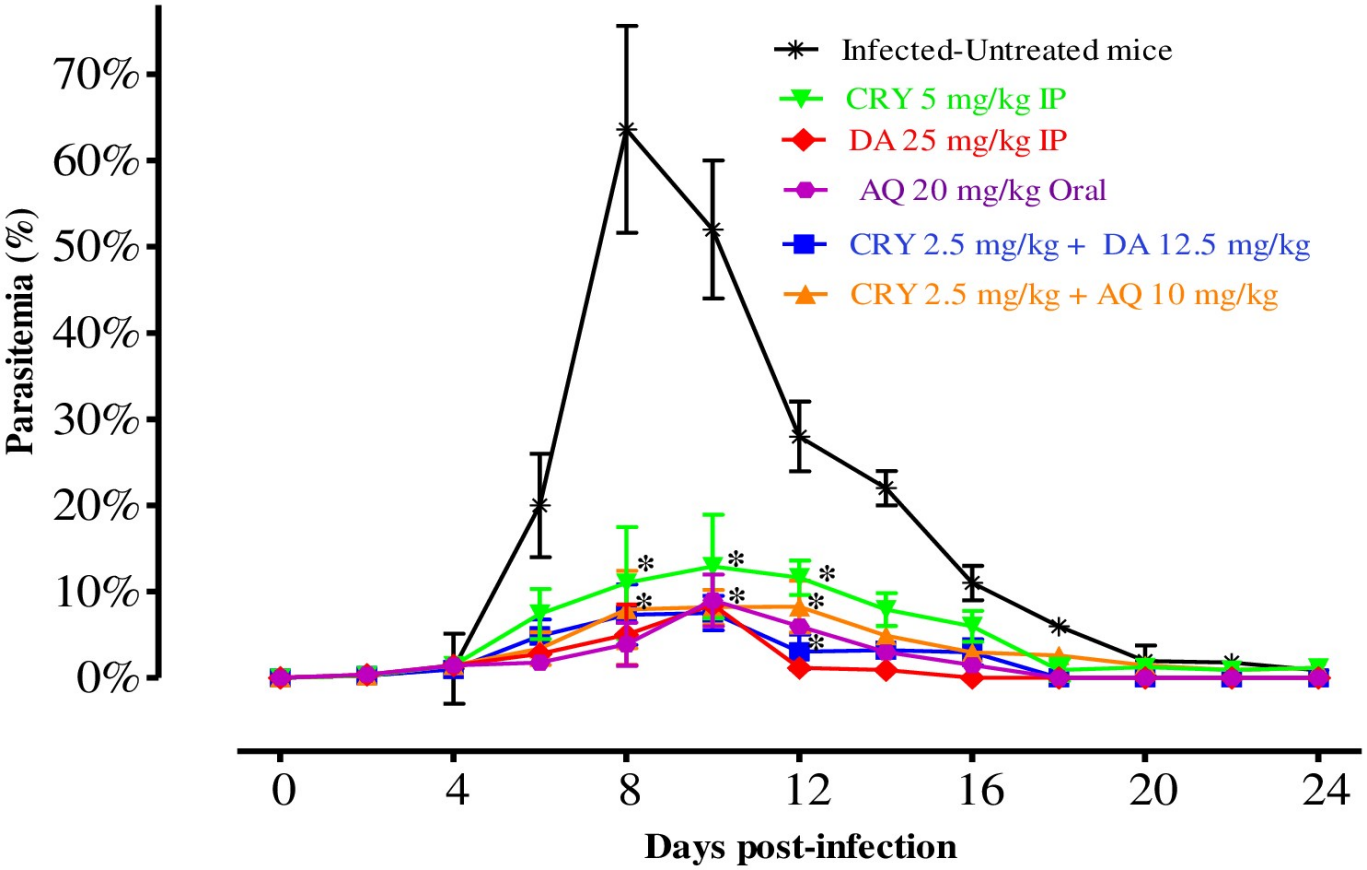
The growth inhibition of CRY on *B*. *microti in vivo*. The inhibitory effects of DA-i.p., AQ-oral, and CRY-i.p. single treatments and combination treatments of CRY with DA and AQ as compared with the untreated group. The asterisks (*) indicate statistical significance (*P* < 0.05) between treated and untreated mice. The arrow indicates 5 consecutive days of treatment. Parasitemia was calculated by counting infected RBCs among 5000 RBCs using Giemsa-stained thin blood smears.

The inhibitory effect of CRY was further evaluated on the multiplication of *B*. *microti*-infected mice as compared with other drugs (Fig 3).

**Fig 4:**
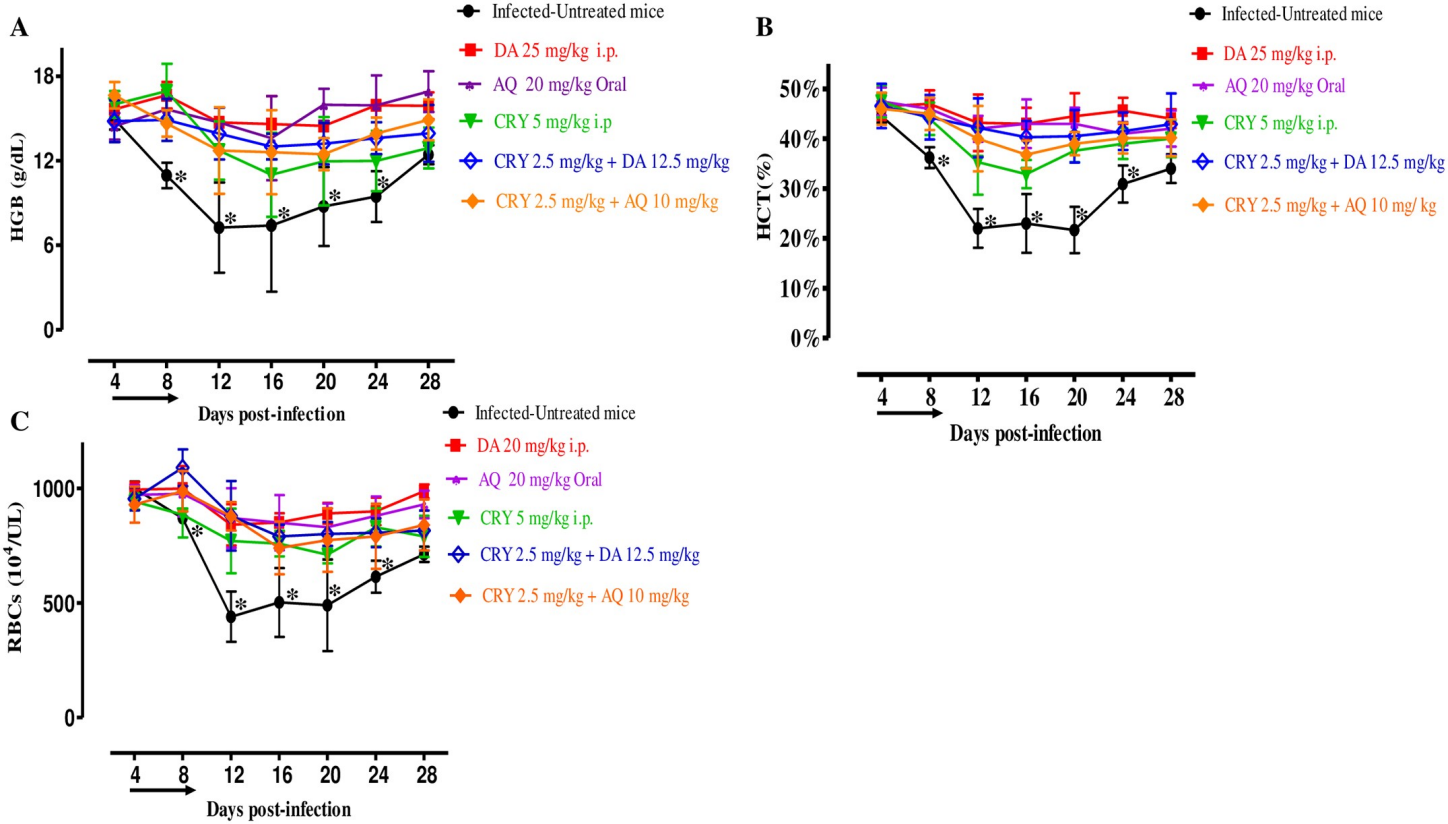
Hematology profiles of CRY-treated mice *in vivo*. Graphs showing the changes in the HGB (A), HCT (B), and RBCs (C) of treated mice as compared with untreated mice. The values plotted are the mean ± standard deviation for two separate trials. Asterisks (*) indicate statistical significance (*P* < 0.05) based on the unpaired *t*-test analysis. The arrow indicates 5 consecutive days of treatment.

In the DMSO control group, the multiplication of *B*. *microti* increased significantly and reached the highest parasitemia at 62.6% on day 8 p.i. In each treated group, the level of parasitemia was cleared at a significantly lower percent parasitemia than the control group (ANOVA; *F*_(2.645,11.21)_ = 5.374, *P* = 0.003 for monotherapy; and ANOVA; *F*_(2.845,13.72)_ = 5.023, *P* < 0.0001 for combination treatment) from day 6 to 12 p.i. In mono-chemotherapy-treated mice, the peak parasitemia level reached 12.9, 8.5, and 9% on day 10 in 5 mg/kg CRY, 25 mg/kg DA, and 20 mg/kg AQ, respectively (Fig 3). In mice treated with 25 mg/kg DA and 20 mg/kg AQ, parasitemia was undetectable via microscopy starting on day 16 and 18 p.i., respectively. In mice treated with 5 mg/kg of CRY, parasitemia was undetectable by microscopic examination on day 28 p.i., while in the combination chemotherapy-treated groups, the peak parasitemia level was lower than that with single treatment at higher doses and reached 7.5 and 8% in 2.5 mg/kg CRY-12.5 mg/kg DA and 2.5 mg/kg CRY-10 mg/kg AQ on day 10, respectively (Fig 3). Additionally, parasite clearance was faster than that of CRY single treatment, whereas parasitemia was undetectable via microscopy assay in mice treated with 2.5 mg/kg CRY-12.5 mg/kg DA and 2.5 mg/kg CRY-10 mg/kg AQ on days 18 and 26 p.i., respectively. Moreover, *B*. *microti* infection diminished HGB percentage, hematocrit (HCT) count, and RBC count in mouse blood, as observed in the DMSO control group on days 8, 12, 16, and 20 p.i. Bodyweight of treated and untreated mice did not show any significant difference, while HGB percentage (ANOVA; *F*_(4.523, 15.71)_ = 16.14, *P* < 0.0001), HCT (ANOVA; *F*_(5.945, 14.92)_ = 11.77, *P* < 0.0001), and RBC count (ANOVA; *F*_(6.052, 13.88)_ = 28.95, *P* < 0.0001) were observed to be significantly different between the DMSO control group and all drug-treated groups on days 8, 12, 16, and 20 (Fig 4A–4C).

**Fig 5:**
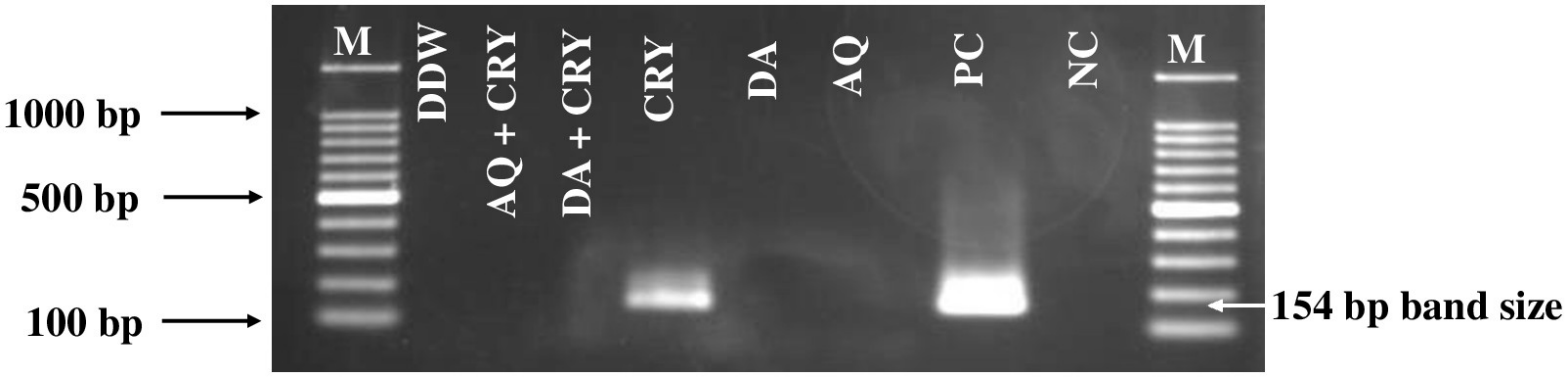
Molecular detection of parasite DNA in the blood of treated groups. The image shows the molecular detection of parasites in the blood of treated groups on day 49. M indicates the marker, NC indicates the untreated-uninfected group used as a negative control, and PC indicates the untreated-infected group used as the positive control. The arrow shows the expected band length of 154 bp for positive cases of *B*. *microti*.

Parasite DNA was not detected in 25 mg/kg DA, 20 mg/kg AQ, 2.5 mg/kg CRY-12.5 mg/kg DA, 2.5 mg/kg CRY-10 mg/kg AQ on day 49, while parasite DNA was still detected until day 49 in the 5 mg/kg CRY group (Fig 5).

## Discussion

The emergence of resistance, residue, and toxicity problems of the current antibabesial drugs promotes a renewed effort to seek alternative therapies to meet these challenges. Today, drugs of herbal origin are considered safe for use in human and animal medicine, as they are easily metabolized compared to synthetic chemicals [20]. In the same way, combination therapy is considered to be the preferred approach to addressing these problems, as it seeks to reduce or delay the development of resistance, as well as to reduce toxicity by using lower doses [21]. CRY is a well-known antimalarial compound that exhibits considerable synergy with traditional antimalarial agents and has demonstrated strong gametocidal activity against *P*. *falciparum*. Moreover, CRY and its analogues are therefore being identified as potential antimicrobial agents [5]. Notably, several studies show that CRY and its analogues can be a source of new lead molecules for cancer and antimalarial chemotherapy [13, 18].

This study reveals the potent inhibitory effect of CRY against four *Babesia* species and *T*. *equi in vitro* and *B*. *microti in vivo*. Recently, CRY has been documented to strongly inhibit the growth of *Plasmodium*, the closely related apicomplexan parasite [13], and also inhibited the growth of *Trypanosoma* and *Leishmania* by a different mode of actions [22, 23]. Lavrado et al. reported that CRY derivatives strongly inhibited *Trypanosoma brucei* by inhibiting parasitic papain-like cysteine proteases [22], while Lisgarten et al. documented that the antitrypanosomal activity of CRY might be through DNA intercalations [19]. Meanwhile, Sengupta et al. documented CRY’s potent inhibitory effect on *Leishmania donovani* strain AG83 promastigotes, whereas CRY increased the production of cellular ROS with a concomitant decrease in cellular glutathione (GSH) levels and an increase in the level of lipid peroxidation [24]. Onyeibor et al. reported that CRY has potent *in vitro* antiplasmodial activity due to its ability to intercalate with DNA and inhibit topoisomerase II as well as DNA synthesis [25]. However, Wright et al. documented that the antiplasmodial action of CRY is due to a chloroquine-like action [12]. Recently, Forkuo et al. reported that CRY is likely to extensively distribute in humans, accumulate in vital organs, and prolong its residence time in plasma, resulting in prolonging the CRY’s elimination half-life (t_1/2_) [26]. This property would be particularly advantageous for the clearance of parasites’ erythrocytic stage. Although there are close similarities between *Plasmodium* and *Babesia* and *Theileria* parasites, CRY’s activity against piroplasm parasites is biologically interesting and yet uncertain as these parasites do not degrade hemoglobin, suggesting that the primary mode of CRY’s anti-babesia action is distinct from its anti-plasmodial action. Compared to current data, the mode of action is still not well understood. However, more research is required to verify the exact mechanism of action of CRY against *Babesia* and *Theileria*.

Our cytotoxicity study on NIH/3T3, HFF, and MDBK cell lines showed that the selectivity index of CRY was slightly high among the tested species, which means that CRY was more likely to affect the viability of *Babesia* and *Theileria* than the host cells. Recently, Pal and Katiyar. reported that CRY is highly selective in inhibiting the viability of skin cancer cells versus normal cells [18].

Interestingly, combination treatment has been recommended against drug-resistant protozoal and bacterial pathogens. Since, the cytotoxicity study revealed that CRY resulted in lower selective index than DA. Therefore, the combination study aimed to enhance the efficacy of CRY while reducing the dose that led to reduced toxicity, subsequently reducing their toxic side effects. The existing study evaluated the potency of CRY as an inhibitor in combination with DA, AQ, and CF. Synergetic and additive effects were found between CRY and AQ against all species, while CRY showed an additive effect with DA against *B*. *bovis*, *B*. *caballi*, and *T*. *equi*. These findings are consistent with those of Forkuo et al., which found that the combination of CRY and artemisinin had a synergistic effect, both *in vivo* and *in vitro*, against *Plasmodium berghei* NK-65 and *P*. *falciparum* 3D7, without causing acute toxicity, as no significant changes in histopathology or biochemical and hematological parameters were observed in healthy Sprague-Dawley [13]. Therefore, CRY and AQ combinations could be used as treatment options for human babesiosis.

The potent effect of CRY combined with DA or AQ was also observed on the multiplication of *B*. *microti* in a mouse model. CRY administered i.p. showed a high chemotherapeutic effect against *B*. *microti* with no apparent adverse effects in mice. This finding is compatible with that of Wright et al., who reported that CRY suppressed *P*. *berghei* parasitemia in mice by 80.5% with no apparent toxicity to the mice [12]. Interestingly, combination treatment of CRY-DA and -AQ at a half dose showed potent chemotherapeutic effect comparable to that showed by single drugs at full doses, emphasizing that CRY is a good combinatorial drug. Hematology profiles (RBCs count, HGB, and HCT) and parasitemia were also improved as compared with those of the DMSO control group. With reference to the above, CRY might be safe for use in humans and animals.

The PCR assay performed on blood samples collected on day 49 p.i. confirmed the absence of *B*. *microti* DNA in CRY-DA and CRY-AQ combination-treated groups. These findings are consistent with those shown by Forkuo et al., who documented that combination treatment with CRY and artemisinin is more effective in eliminating parasitemia than CRY alone. They reported that this combination therapy did not show acute toxicity as no major changes were observed in biochemical, histopathology and the haematological parameters in healthy Sprague-Dawley rats [26]. Moreover, the three-day anti-malarial combination therapy offers a better choice for rapid clearance of parasites in the blood compared to single treatment. Moreover, Ameyaw et al., indicating that cryptolepine and xylopic acid combined therapy yielded a synergistic anti-plasmodial effect without showing any significant adverse effects on the spleen, kidney, and liver, while the testis were affected at high doses. The only possible explanation is that this increase in *in vivo* efficacy is the result of synergy between CRY and artemisinin or xylopic acid or a prolonged half-life of one or both compounds, and therefore this combination therapy is predicted to result in inhibiting hemozoin biosynthesis or cause its degradation [27]. Interestingly, combination treatment of CRY and DA *in vitro* showed a moderate antagonistic effect against two *Babesia* species; *B*. *bigemina* and *B*. *divergens*, our *in vivo* and PCR results showed high chemotherapeutic efficacy and parasite clearance with this combination against *B*. *microti* in mice. El Kouni. documented that many drugs have shown varying efficacy against different species of parasites; this may be due to different nutritional requirements for parasite growth, as well as the basic biochemistry, physiology, and molecular biology of parasites and of their interactions with their hosts, including bioavailability and metabolism of administered drugs in the mouse model, which may be the main reason of the difference of the combination effect of CRY with DA *in vitro* and *in vivo* [28]. Taken together, these findings advocate for the anti-piroplasmic potential of CRY against the growth of *Babesia* and *Theileria* parasites that can open the way to search for other highly effective and less toxic synthetic cryptolepine analogs. However, it should be noted that there are some limitations to the present study. Although the CRY-DA and CRY-AQ combinations showed a potential anti-piroplasmic effect *in vitro* and *in vivo*, the reason for this potential inhibitory effect is not fully understood. Thus, further studies are needed to detect the exact mechanism of action of CRY against *Babesia* and *Theileria* so as to better clarify these relationships with other drugs such as DA, CF, and AQ. Moreover, further *in vivo* experiments are required to ensure the full parasite clearance with no recrudescence after treatment has been discontinued. Such findings will set the stage for additional clinical assessment of CRY and DA combination therapy for the treatment of equine and bovine piroplasmosis as well as CRY and AQ combination for the treatment of human babesiosis [29].

In summary, CRY showed a potent growth inhibitory effect against several *Babesia* species and *T*. *equi in vitro* and chemotherapeutic efficacy against *B*. *microti in vivo*. However, we did not perform a viability test in order to assess the parasite viability as reported by Batiha et al., who documented that all tested parasites could not revive even after drug withdrawal [30]. Therefore, a viability assay is required to detect whether the effect of CRY is inhibition of the growth or death in the future study. CRY effectiveness *in vivo* was comparable to that shown by DA and AQ and showed no apparent adverse effects in mice. CRY–DA and CRY–AQ combinations showed higher efficiency against *B*. *microti* in mice than did CRY single drug treatment. Conclusively, CRY would be a promising drug for the treatment of clinical disease caused by *Babesia* and *Theileria* in humans and animals.

## Materials and methods

### Cultivation conditions

#### Ethics statement

All experiments were approved by the Animal Welfare Committee and conducted in accordance with the standards for the care and management of experimental animals as stipulated by Obihiro University of Agriculture and Veterinary Medicine (animal experiment accession number: 29-016-8). These regulations were established by Fundamental Guidelines for Proper Conduct of Animal Experiment and Related Activities in Academic Research Institutions, the Ministry of Education, Culture, Sports and Technology (MEXT), Japan.

#### Chemical reagents

Cryptolepine hydrate (C_16_H_12_N_2_) (CRY; Sigma-Aldrich, Tokyo, Japan) powder was examined for its babesiocidal effects; DA, CF, and AQ (Sigma-Aldrich, Tokyo, Japan) powders were used as comparator drugs *in vitro* and used in combination with CRY either *in vitro* or *in vivo*. A stock solution of 10 mM of all powders was prepared in dimethyl sulfoxide (DMSO) and stored at -30°C until use. A lysis buffer containing Tris-HCl (130 mM at pH 7.5), EDTA (10 mM), saponin (0.016%; w/v), and Triton X-100 (1.6%; v/v) was prepared, filtered through 0.22 μm of polyethersulfone, and stored at 4°C. The lysis buffer was mixed with 0.2 μl/ml SYBR Green I nucleic acid stain (SG1; 10,000×, Lonza America, Alpharetta, Georgia, USA) prior to the fluorescence assay.

#### Mice

Rodent *B*. *microti* (Munich strain) was recovered from -80°C stock and injected intraperitoneally (i.p.) in two 8-week-old female BALB/c mice purchased from CLEA Japan (Clea Japan, Tokyo, Japan) [31]. The mice were euthanized using an anesthesia system containing isoflurane after parasitemia reached approximately 30% and the blood was collected by cardiac puncture [32] to conduct the *in vivo* studies.

#### *In vitro* cultivation of parasites

Texas strain of *B*. *bovis* and Argentina strain of *B*. *bigemina* were grown using bovine red blood cells (RBCs, collected from cattle farm of Obihiro University of Agriculture and Veterinary Medicine and stored at 4°C) in medium 199 (M199; Sigma-Aldrich, Tokyo, Japan) supplemented with 40% bovine serum, while RPMI 1640 medium (Sigma-Aldrich, Tokyo, Japan) was used for *B*. *divergens* (German strain) cultivation [33, 34]. The United States Department of Agriculture (USDA) strain of *T*. *equi* was grown in equine RBCs (collected from horse farm of Obihiro University of Agriculture and Veterinary Medicine and stored at 4°C) in M199 supplemented with 40% equine serum, and hypoxanthine (MP Biomedicals, Santa Ana, CA, USA) at a final concentration of 13.6 μg/ml was used as a vital supplement, while GIT medium was used for *B*. *caballi* (USDA strain) cultivation. Sixty μg/ml of streptomycin, 0.15 μg/ml of amphotericin B, and 60 U/ml of penicillin G (Sigma-Aldrich, Tokyo, Japan) were added to all media to prevent fungal and bacterial contamination. All parasite cultures were maintained for a long time in a microaerophilic stationary-phase culture system [35, 36].

#### The growth inhibitory assay *in vitro*

The inhibition assay was conducted according to the previously described protocol [33]. Briefly, different concentrations of CRY, DA, CF, and AQ were added in triplicate in 96-well plates with 1% parasitemia and 2.5% HCT for *B*. *bovis* and *B*. *bigemina* and 5% HCT for *B*. *divergens*, *B*. *caballi*, and *T*. *equi*. The plates were incubated in a humidified incubator with 5% CO_2_, 5% O_2_, and 90% N_2_ for 96 h without changing the media. After 96 h, 100 μl of lysis buffer containing 2 × SG1 was directly added to each well and the plates were then wrapped in aluminum foil for protection from direct light and incubated for 6 h at room temperature. Afterward, the fluorescence was measured using a fluorescence spectrophotometer (Fluoroskan Ascent, Thermo Fisher Scientific, USA) at 485 nm excitation and 518 nm emission wavelengths. The relative fluorescence value was set to percentages after subtracting the mean values of the negative control. The experiment was repeated thrice.

#### Cell cultures

Madin–Darby bovine kidney (MDBK) (ECACC), mouse embryonic fibroblast (NIH/3T3 ATCC CRL-1658) and Human foreskin fibroblast (HFF HFF-1 ATCC SCRC-1041) cell lines were recovered from -80°C stock and cultured continuously in our laboratory at 37°C in a humidified incubator with 5% CO_2_. In 75 cm^2^ culture flasks, NIH/3T3 and HFF cell lines were maintained with Dulbecco’s Modified Eagle’s Medium (DMEM; Gibco, Grand Island, NY, USA), while the MDBK cell line was maintained with Minimum Essential Medium Eagle (MEM; Gibco). Ten percent fetal bovine serum, 1% glutamine, and 0.5% penicillin/streptomycin (Gibco) were added to all media and the cells were stained with 4, 6-diamidino-2-phenylindole dihydrochloride (DAPI; Sigma-Aldrich, St. Louis, MO, USA) to ensure that they were free from mycoplasma contamination. Afterward, the cells were washing twice with Dulbecco’s phosphate-buffered saline and TrypLE Express (Gibco) was used for cell detachment from the culture flask. Finally, Neubauer improved C-Chip (NanoEntek Inc., Seoul, Korea) was used for counting the viable cells after staining with 0.4% trypan blue solution.

#### Cytotoxicity assay of CRY, DA, CF, and AQ on NIH/3T3, HFF, and MDBK cell lines

The cytotoxicity assay was performed in accordance with the previously described protocol [33, 30]. Briefly, the assay was carried out using 96-well plates at 37 °C in a humidified incubator with 5% CO_2_, 100 μl of cells at a density of 5 x 10^4^ cells/ml was seeded per well and allowed to attach to the plate for 24 h. Ten microliters of twofold drug dilutions was added to each well to a final concentration of 0.781 to 100 μM in triplicate and incubated for another 24 h. Ten μl of Cell Counting Kits-8 (CCK-8; Dojindo, Japan) was added to each well and incubated for another 3 h, and the absorbance was measured at 450 nm using a microplate reader.

#### Effect of CRY on the host RBCs

To investigate the hemolytic effect of CRY on host (bovine or equine) RBCs, bovine and equine RBCs were incubated with 1 and 10 μM of CRY for 3 and 6 h. The RBCs were washed thrice with phosphate-buffered saline (PBS) and mixed with *B*. *divergens* and *B*. *caballi* infected RBCs (iRBCs, recovered from −80 °C stock at our laboratory) harvested with a parasitemia of 8% and 3%, respectively and adjusted to 1% parasitemia with fresh RBCs. In a 24-well plate, 100 μl of iRBCs was added to 900 μl of complete medium and the parasite growth was monitored daily by microscopic examination of a Giemsa-stained blood smear for 4 days.

#### The combination treatment of CRY with DA, CF, and AQ *in vitro*

Drug combination assay for CRY with DA, CF, and AQ was performed at the same time with the single drug assay at a constant ratio (1:1) in accordance with the Chou-Talalay method [37]. Briefly, in the same plate with a single drug inhibition assay, two-drug combinations of CRY + DA, CRY + CF, and CRY + AQ at five different concentrations of 0.25 × IC_50_, 0.5 × IC_50_, IC_50_, 2 × IC_50_, and 4 × IC_50_ (S2 Table) were added in triplicate to the wells containing iRBCs. The plates were incubated in a humidified incubator with 5% CO_2_, 5% O_2_, and 90% N_2_ for 4 days without changing medium. After 4 days, 100 μl of lysis buffer containing 2 × SG1 was added to each well, and the fluorescence values were measured as mentioned above.

#### The chemotherapeutic effect of CRY against *B*. *microti*

The promising effect of CRY *in vitro* encouraged us to evaluate its *in vivo* effects against *B*. *microti* in mice. The assay was conducted in accordance with the previously described protocol [6]. In two separate trials, 35 female 8-week-old BALB/c mice, housed under a pathogen-free environment with controlled humidity, temperature (22°C) and a 12 h light/ dark cycle were caged in seven groups, each group consisting of five mice. Six groups were i.p. injected with 0.5 ml of inoculum (1×10^7^ *B*. *microti* iRBCs), while the seventh group was left uninfected and untreated to act as a negative control. When the average parasitemia in all infected mice reached approximately 1%, the 5-day drug treatment was initiated. The first and second groups were i.p. injected with 25 mg/kg body weight (BW) of DA and 5 mg/kg BW of CRY, respectively. The third group received 20 mg/kg BW of AQ orally, while the fourth and fifth groups were administered a combination of 2.5 mg/kg BW CRY + 12.5 mg/kg BW DA and 2.5 mg/kg BW CRY + 10 mg/kg AQ via a route similar to that of the single-drug treatment, respectively. The sixth group received 5% DMSO in physiological saline i.p. as a positive control. Parasitemia and hematology profiles (RBCs, hemoglobin (HGB), and HCT) were monitored every 2 and 4 days by microscopic examination and hematology analyzer (Celltac α MEK-6450, Nihon Kohden Corporation, Tokyo, Japan), respectively. On day 49, all mice were euthanized using an anesthesia system containing isoflurane and the blood was collected by cardiac puncture for PCR detection of the parasites.

#### Genomic DNA extraction and PCR detection of *B*. *microti*

The nested PCR (nPCR) that targeted the *B*. *microti* small-subunit rRNA (ss-rRNA) gene was performed in accordance with the previously described protocol [6] and the genomic DNA was extracted from blood samples collected from all mice groups on day 49 post-infection (p.i.) using a QIAamp DNA Blood Mini Kit (Qiagen, Tokyo, Japan). PCR cycling conditions were performed as previously described [6] and the bands with an expected size of 154 bp were considered positive.

#### Statistical analysis

The IC_50_ values of CRY, DA, CF, and AQ were determined using the nonlinear regression curve fit in GraphPad Prism (GraphPad Software Inc., San Diego, CA, USA). Combination index (CI) values for the drug combination were calculated using CompuSyn software [37], and the degree of synergism was determined as the weighted average of CI values using a formula [(1 x IC_50_) + (2 x IC_75_) + (3 x IC_90_) + (4 x IC_95_)]/10 [30]. The differences in parasitemia and hematology profiles in the *B*. *microti*–infected mice were analyzed using an independent Student’s *t*-test and one-way ANOVA test using GraphPad Prism version 5.0 for Windows (GraphPad Software Inc., San Diego, CA, USA). A *P*-value < 0.05 was considered statistically significant.

## Supporting information

S1 Table The IC_50_ and selectivity indices of the control drugs (AQ and CF). (DOCX)

S2 Table Concentrations of CRY combined with DA, AQ, and CF against four *Babesia* species and *T*. *equi in vitro*. (DOCX)
