# Supplementary material for: In vitro and in vivo growth inhibitory activities of cryptolepine hydrate against several Babesia species and Theileria equi
Source: PLoS Negl Trop Dis. 2020 Aug 27;14(8):e0008489. doi: 10.1371/journal.pntd.0008489 (PMC7451656; doi:10.1371/journal.pntd.0008489)
Supplement: S1 Table — (DOCX) [file pntd.0008489.s001.docx]

**S1 Table. The IC_50_ and selectivity index of AQ and CF**

| **Compound** | ***Babesia* and *Theileria*** | **IC_50_ ( nM )^a^** | **EC_50_ ( µM )^b^** | | | **Selective index^c^** | | |
| --- | --- | --- | --- | --- | --- | --- | --- | --- |
|  |  |  | **MDBK** | **NIH/3T3** | **HFF** | **MDBK** | **NIH/3T3** | **HFF** |
| **AQ** | *B. bovis* | **39 ± 0.002** | **˃100** | **˃100** | **˃100** | **˃ 2564.1** | **˃ 2564.1** | **˃ 2564.1** |
|  | *B. bigemina* | **701 ± 0.04** | **˃100** | **˃100** | **˃100** | **˃ 142.7** | **˃ 142.7** | **˃ 142.7** |
|  | *B. divergens* | **38 ± 0.002** | **˃100** | **˃100** | **˃100** | **˃ 2631.6** | **˃ 2631.6** | **˃ 2631.6** |
|  | *B. caballi* | **102 ± 0.014** | **˃100** | **˃100** | **˃100** | **˃ 980.4** | **˃ 980.4** | **˃ 980.4** |
|  | *T. equi* | **95 ± 0.065** | **˃100** | **˃100** | **˃100** | **˃ 1052.6** | **˃ 1052.6** | **˃ 1052.6** |
| **CF** | *B. bovis* | **8240 ± 1.7** | **34.72 ± 3.4** | **˃100** | **˃100** | **4.2** | **˃ 12.1** | **˃ 12.1** |
|  | *B. bigemina* | **5730 ± 1.9** | **34.72 ± 3.4** | **˃100** | **˃100** | **6.1** | **˃ 17.5** | **˃ 17.5** |
|  | *B. divergens* | **13850 ± 4.3** | **34.72 ± 3.4** | **˃100** | **˃100** | **2.5** | **˃ 7.2** | **˃ 7.2** |
|  | *B. caballi* | **7950 ± 1.8** | **34.72 ± 3.4** | **˃100** | **˃100** | **4.4** | **˃ 12.6** | **˃ 12.6** |
|  | *T. equi* | **2880 ± 0.9** | **34.72 ± 3.4** | **˃100** | **˃100** | **12.1** | **˃ 34.7** | **˃ 34.7** |

^a^Half-maximal inhibition concentrations of atovaquone (AQ) and clofazimine (CF) on the *in vitro* culture of parasites. The value was determined from the dose-response curve using nonlinear regression (curve fit analysis). The values are the means of triplicate experiments.

^b^Half-maximal effective concentration of AQ and CF on the cell line. The values were determined from the dose-response curve using nonlinear regression (curve fit analysis). The values are the means of triplicate experiments.

^c^Ratio of the EC_50_ of cell lines to the IC_50_ of each species. High numbers are favorable.
