# Supplementary material for: In vitro and in vivo growth inhibitory activities of cryptolepine hydrate against several Babesia species and Theileria equi
Source: PLoS Negl Trop Dis. 2020 Aug 27;14(8):e0008489. doi: 10.1371/journal.pntd.0008489 (PMC7451656; doi:10.1371/journal.pntd.0008489)
Supplement: S2 Table — (DOCX) [file pntd.0008489.s002.docx]

**S2 Table. Concentrations of CRY combined with DA, AQ, and CF against *Babesia* and *Theileria* parasites *in vitro***

Note: ^a^C_1_–C_5_ refers to the concentrations (**µM**) 0.25 × IC_50_, 0.5 × IC_50_, 1 × IC_50_, 2 × IC_50_, 4 × IC_50_ of cryptolepine (CRY) combined with diminazene aceturate (DA), atovaquone (AQ), and clofazimine (CF). Combined concentrations were based on the calculated IC_50_ values obtained from the *in vitro* fluorescence-based assay.

| **Parasite** | **Concentration**  (**µM**) | **CRY** | **DA** | **AQ** | **CF** |
| --- | --- | --- | --- | --- | --- |
| ***B. bovis*** | **C_1_** | 0.435 | 0.0875 | 0.00975 | 2.06 |
|  | **C_2_** | 0.87 | 0.175 | 0.0195 | 4.12 |
|  | **C_3_** | 1.74 | 0.35 | 0.039 | 8.24 |
|  | **C_4_** | 3.48 | 0.7 | 0.078 | 16.48 |
|  | **C_5_** | 6.96 | 1.4 | 0.156 | 32.96 |
|  |  |  |  |  |  |
| ***B. bigemina*** | **C_1_** | 0.35 | 0.17 | 0.17525 | 1.4325 |
|  | **C_2_** | 0.7 | 0.34 | 0.3505 | 2.865 |
|  | **C_3_** | 1.4 | 0.68 | 0.701 | 5.73 |
|  | **C_4_** | 2.8 | 1.36 | 1.402 | 11.46 |
|  | **C_5_** | 5.6 | 2.72 | 2.804 | 22.92 |
|  |  |  |  |  |  |
| ***B. divergens*** | **C_1_** | 0.1945 | 0.1075 | 0.0095 | 3.4625 |
|  | **C_2_** | 0.389 | 0.215 | 0.019 | 6.925 |
|  | **C_3_** | 0.778 | 0.43 | 0.038 | 13.85 |
|  | **C_4_** | 1.556 | 0.86 | 0.076 | 27.7 |
|  | **C_5_** | 3.112 | 1.72 | 0.152 | 55.4 |
|  |  |  |  |  |  |
| ***B. caballi*** | **C_1_** | 0.15 | 0.0055 | 0.0255 | 1.9875 |
|  | **C_2_** | 0.3 | 0.011 | 0.051 | 3.975 |
|  | **C_3_** | 0.6 | 0.022 | 0.102 | 7.95 |
|  | **C_4_** | 1.2 | 0.044 | 0.204 | 15.9 |
|  | **C_5_** | 2.4 | 0.088 | 0.408 | 31.8 |
|  |  |  |  |  |  |
| ***T. equi*** | **C_1_** | 0.1825 | 0.775 | 0.02375 | 0.72 |
|  | **C_2_** | 0.365 | 0.355 | 0.0475 | 1.44 |
|  | **C_3_** | 0.73 | 0.71 | 0.095 | 2.88 |
|  | **C_4_** | 1.46 | 1.42 | 0.19 | 5.76 |
|  | **C_5_** | 2.92 | 2.84 | 0.38 | 11.52 |
